# Poor sleep quality in patients with multiple sclerosis: gender differences

**DOI:** 10.1002/brb3.553

**Published:** 2016-09-20

**Authors:** Marianna Vitkova, Jaroslav Rosenberger, Zuzana Gdovinova, Jarmila Szilasiova, Pavol Mikula, Johan W. Groothoff, Sijmen A. Reijneveld, Jitse P. van Dijk

**Affiliations:** ^1^ Department of NeurologyFaculty of MedicineSafarik UniversityKosiceSlovakia; ^2^ Graduate School Kosice Institute for Society and HealthSafarik UniversityKosiceSlovakia; ^3^ Department of Community & Occupational HealthUniversity of GroningenUniversity Medical Center GroningenGroningenthe Netherlands

**Keywords:** gender differences, multiple sclerosis, sleep quality

## Abstract

**Objectives:**

Most of the psychological and physical factors associated with poor sleep quality in patients with multiple sclerosis (MS) have a different prevalence in women and men, but whether or not these factors contribute differently to sleep quality in women and men with MS remains unclear. The aim of this study was to identify possible gender differences in factors related to poor sleep quality in MS patients.

**Material and Methods:**

We collected data from 153 patients with MS. Patients filled out the Pittsburgh Sleep Quality Index (PSQI), the Hospital Anxiety and Depression Scale, and one item of the Short Form‐36 regarding pain.

**Results:**

The best model of predictors of poor sleep quality consisting of gender, depression, anxiety, pain, and the interaction between gender and pain showed that the only variable interacting with gender, which was significantly associated with poor sleep quality was pain (odds ratio [OR] for interaction of pain with male gender was 15.4, 95% CI: 2.4; 39.5). Separate models for men and women consisting of pain, depression, anxiety, after adjustment for age, disease duration, and disability showed that pain was the only variable associated with poor sleep quality in men (OR = 12.7, 95% CI: 1.9; 29.6), whereas depression (OR = 4.1, 95% CI: 1.3; 13.2) and anxiety (OR = 6.8, 95% CI: 2.4; 19.1) were in women.

**Conclusions:**

Factors contributing to poor sleep quality in MS patients differ by gender. Depression and anxiety are associated with poor sleep quality in women, whereas pain is in men. This highlights the need to apply gender‐specific approaches to the treatment of sleep disorders.

## 1 Introduction

Most patients with multiple sclerosis (MS) complain of poor sleep quality (Vitková, Gdovinova, & Rosenberger, 2014), which frequent interferes with their daily activities and worsens their health‐related quality of life (Merlino, Fratticci, & Lenchig, 2009). MS patients with poor sleep complain of excessive daytime sleepiness, impaired concentration, memory and learning deficits, altered mood, and fatigue (Schutte‐Rodin, Broch, & Buysse, 2008; Kaminska, Kimoff, & Benedetti, 2012). Merlino et al. (2009) showed that poorer sleep quality was directly correlated with a higher disability as measured by EDSS (the expanded disability status scale).

The prevalence of sleep problems in the MS population ranges from 47 to 62%, with a higher prevalence in women (Vitková et al., 2014; Lobentanz, Asenbaum, & Vass, 2004). Boe Lunde et al., (2012 found that women also had much poorer sleep quality in a sample of 90 patients with MS (Boe Lunde, Aae, & Indrevag, 2012). A similar woman predisposition for poorer sleep quality has also been found in studies of otherwise healthy people (Mallampalli & Carter, 2002; van den Berg, Miedema, & Tulen, 2009), and these studies have proposed several explanations for this finding. First, sex hormones and genetic mechanisms may contribute to sleep differences between women and men (Silveyra, Cataldi, Lux‐Lantos, & Libertun, 2009; Zhang & Wing, 2006). Second, gender differences may exist in psychosocial factors that affect sleep quality (Mallampalli & Carter, 2002; van den Berg et al., 2009). More specifically, the prevalence of anxiety and/or depression is consistently found to be higher in women, which may lead to poorer sleep quality in women than in men. Third, some physical factors that disrupt sleep, such as pain, are again more prevalent in female subjects (Zhang & Wing, 2006).

Factors associated with poor sleep quality in MS patients are very similar to those reported in the general population. A higher level of depression and/or anxiety, increased fatigue, and more severe pain or bladder dysfunction have all been reported as important contributors of poor sleep (Vitková et al., 2014; Merlino et al., 2009; Boe Lunde et al., 2012). Studies in the general population (Mallampalli & Carter, 2002) or in patients with other chronic disorders (Woosley, Lichtstein, & Taylor, 2012) explained the role of gender in sleep quality mostly by different prevalence rates of factors contributing to poor sleep in women and men. The conclusions of those studies cannot be clearly translated to the MS population, however. Research findings in these patients are rather ambiguous regarding gender differences in the prevalence of factors associated with sleep quality in MS patients. They seem to be different from those reported for the general population and for other chronic disorders (Mallampalli & Carter, 2002; Woosley et al., 2012).

Regarding the higher prevalence of depression/anxiety in women, some studies confirmed such an association for MS patients (Patten, Beck, & Williams, 2003), whereas others did not (Stordal, Bjartveit Kruger, & Dahl, 2001; Dahl, Stordal, Lydersen, & Midgard, 2009). Research on gender differences in the prevalence of pain among patients with MS is very scarce and findings are conflicting. Some studies reported an association between pain and gender (Moulin, Foley, & Ebers, 1988; Hadjimichael, Kerns, & Rizzo, 2007), whereas others found no gender differences in the prevalence of pain (Svendsen, Jensen, & Overvad, 2003; Archibald, McGrath, & Ritvo, 1994). Regarding fatigue, previous research has failed to show any association between fatigue and gender in MS patients (Anens, Emtner, & Zetterberg, 2014).

To the best of our knowledge there is no study exploring whether the predictors of poor sleep quality are different in women and men with multiple sclerosis. Thus, the aim of our study is to identify possible gender differences in psychological and physical factors related to poor sleep quality in patients with MS.

## 2 Materials and Methods

### 2.1 Sample and procedure

The study comprised 213 consecutive patients with MS from the eastern part of Slovakia. Patients were recruited from our clinical MS database between September 2011 and November 2013. All patients were diagnosed according to the McDonalds criteria (2010). Of these, 60 patients refused to participate (response rate of 72%) in the study. Patients were to be excluded if they had a cognitive dysfunction determined by a Mini‐Mental State Examination (MMSE) score of <24 or a history of psychiatric or medical conditions affecting the outcomes of the study. However, no patients were excluded because of the exclusion criteria. The final sample thus consisted of 153 patients. The local Ethics Committee of the Faculty of Medicine at PJ Safarik University in Kosice approved the study in 2009. All patients provided written informed consent prior to the study.

An invitation letter, the questionnaires, a written informed consent form, and a nonresponse sheet were sent by postal mail to patients with MS. After 2 weeks a trained interviewer called each patient to find out whether or not the patient agreed to participate in the study. Those who agreed were invited for a face‐to‐face interview enabling clarification of the patient's responses and completion of missing answers in the questionnaires. After this interview, a neurological examination was performed by a single neurologist (Vitková et al., 2014).

### 2.2 Measures

Questionnaires regarding sleep quality, depression, anxiety, and pain were translated from the original language into Slovak. A back‐translation was then made to ensure that no meaning was lost in the original translation, with final changes in the translated version made accordingly (Nagyova, 2009).

#### 2.2.1 Sleep quality

Sleep quality was assessed using the Pittsburgh Sleep Quality Index (PSQI). The PSQI is a self‐rated questionnaire which consists of 19 individual items generating 7 component scores: subjective sleep quality, sleep latency, sleep duration, habitual sleep efficiency, sleep disturbances, use of sleeping medication, and daytime dysfunction (Buysse, Reynolds, & Monk, 1989). After recoding, each component has a possible score of 0–3, where a higher score indicates a greater sleep problem. The global PSQI score is the sum of all components scores (range: 0–21); a score ≥5 represents poor sleepers; <5 represents patients with normal sleep quality (Buysse et al., 1989). The PSQI assesses sleep quality and disturbances over a 1‐month time interval. Cronbach's alpha was 0.87 in our sample.

#### 2.2.2 Psychological and physical factors

*Depression* and *anxiety* were assessed using the self‐administered Hospital Anxiety and Depression Scale (HADS) questionnaire. The HADS consists of two subscales: one for assessing anxiety (HADS‐A) and the other for depression (HADS‐D). Each subscale consists of seven items, with scoring from 0 (no problem) to 3 (extreme problem) (Zigmond & Snaith, 1991). The summary score for both subscales ranges from 0 to 21, with a higher score indicating a worse condition. We applied as cut‐off: a score of 7 or lower indicates noncases, higher than 7 possible and definite cases (Zigmond & Snaith, 1991). In our sample Cronbach's alpha was 0.85 for the depression and 0.86 for the anxiety subscale.

*Pain* was measured by one item of the SF36 (Ware & Sherbourne, 1992): “In the past months, how intense was your pain?” The score ranges from 1 (no pain) to 6 (very severe pain), with a higher score indicating more severe pain (Ware & Sherbourne, 1992).

#### 2.2.3 Covariates

*Sociodemographic* and *clinical data* about the participants, including gender, age, and disease duration, were obtained from structured interview and medical records.

*Disability assessment*. The Expanded Disability Status Scale (EDSS) was used to rate neurological disability (Kurtzke, 1983). The EDSS quantifies disability in eight functional systems: pyramidal, cerebellar, brain stem, sensory, bowel and bladder, visual, cerebral (mental), and others. The EDSS scale ranges from 0 (normal neurological examination) to 10 (death caused by MS). EDSS scores from 1.0 to 4.5 refer to people with MS who are fully ambulatory. EDSS steps 5.0–9.5 are defined by the impairment to walking (Kurtzke, 1983).

### 2.3 Statistical analyses

Firstly, we described the characteristics of the sample (sleep quality, anxiety, depression, pain, disease duration, age, disease course, and EDSS) by gender. The statistical significance of gender differences were tested by *T*‐tests, Mann–Whitney *U*‐tests, and Chi‐Square tests. Next, we computed bivariate correlations among anxiety, depression, pain, disease duration, age, disease course, EDSS, and sleep quality in order to select variables with a statistically significant association with sleep quality for further analysis. Binary logistic regression analyses were then performed to assess the association of pain, depression, and anxiety with sleep quality, including the interactions between gender and these variables, leading to odds ratios (OR) with 95% confidence intervals (95% CI) and *p*‐values. The analyses were adjusted for age, disease duration, and EDSS. Finally, binary logistic regression analyses were performed on the same variables, stratified by gender. Statistical analyses were performed using IBM SPSS 23.0 for Windows.

## 3 Results

A basic description of the MS sample is given in Table 1. The sample consisted of 37 men (mean age 42.5 ± 10.7 and mean disease duration 8.0 ± 5.3) and 116 women (mean age 39.2 ± 9.6 and mean disease duration 7.4 ± 5.5). There were no statistically significant differences in the studied variables between gender groups, except that women suffered significantly more of the relapse‐remitting form of MS.

**Table 1:** Background and clinical characteristics of the sample by gender

** **	All	Men	Women	*p*‐value
No. of patients; *n* (%)	153	37 (24.0)	116 (76.0)	
Disease duration; mean, SD (range)	7.54 ± 5.4 (1–28)	8.0 ± 5.3 (1–19)	7.4 ± 5.5 (1–28)	.80
Age (years); mean, SD (range)	40.0 ± 10.0 (18–61)	42.5 ± 10.7 (18–61)	39.2 ± 9.6 (21–59)	.08
Clinical course				**<.001**
Relapse‐remitting; *n* (%)	122 (80.3)	22 (60.0)	100 (87.0)	
Secondary progressive; *n* (%)	30 (19.7)	15 (40.0)	16 (13.0)	
EDSS; mean, SD (range)	3.1 ± 1.3 (1.0–8.0)	3.5 ± 1.6 (1.0–8.0)	3.0 ± 1.3 (1.5–7.5)	.05
PSQI; mean, SD (range)	5.8 ± 3.5 (0–16)	5.3 ± 3.6 (0–16)	6.0 ± 3.5 (0–16)	.30
Poor sleep (PSQI>5); *n* (%)	68 (44.4)	14 (37.8)	54 (46.6)	.35
HADS‐anxiety; mean, SD (range)	6.8 ± 4.3 (0–17)	6.2 ± 4.1 (0–17)	7.0 ± 4.3 (0–17)	.33
Anxiety (HADS‐A > 7); *n* (%)	68 (44.4)	14 (37.8)	54 (47.7)	.35
HADS‐depression; mean, SD (range)	5.7 ± 4.2 (0–19)	6.0 ± 4.0 (0–17)	5.6 ± 4.3 (0–19)	.47
Depression (HADS‐D > 7); *n* (%)	49 (32.2)	14 (37.8)	35 (30.4)	.88
Pain (SF‐36); mean, SD (range)	2.8 ± 1.3 (1–5)	3.1 ± 1.4 (1–5)	2.7 ± 1.3 (1–5)	.11
Pain>1; *n* (%)	121 (80.0)	19 (75.0)	83 (80.0)	.56

EDS*,* expanded disability status scale; PSQI *,* Pittsburgh sleep quality index; HADS‐D*,* hospital anxiety and depression scale – depression subscale; HADS‐A*,* hospital anxiety and depression scale – anxiety subscale; SF‐36 – short‐form health survey. Bold values indicate the significance p < 0.05.

### 3.1 Gender differences in predictors of poor sleep quality

The best model of predictors of poor sleep quality consisted of gender, depression, anxiety, pain, and the interactions between gender and pain, depression and anxiety. The only variable which significantly interacted with gender was pain (OR for interaction of pain with male gender was 15.4, 95% CI: 2.4; 39.5) (Table 2).

**Table 2:** Factors associated with poor sleep; odds ratios, 95% confidence intervals (CI) for odds ratios (OR) and *p*‐values from binary logistic regression

	PSQI	
	OR (95% CI)	*p*‐value
Predictors		
Male gender (vs. female)	0.1 (0.02; 0.6)	**.012**
Depression (HADS‐D)	5.7 (1.6; 20.2)	**.007**
Anxiety (HADS‐A)	7.0 (2.4; 20.4)	**.001**
Pain (SF‐36)	2.0 (1.2; 3.6)	**.046**
Male gender*pain (SF‐36)	15.4 (2.4; 39.5)	**.005**
Male gender*depression (HADS‐D)	1.1 (0.3; 4.2)	.90
Male gender*anxiety (HADS‐A)	0.6 (0.2; 1.8)	.41

PSQI, Pittsburgh sleep quality index; HADS‐D, hospital anxiety and depression scale – depression subscale; HADS‐A*,* hospital anxiety and depression scale – anxiety subscale; SF‐36*,* short‐form health survey. Bold values indicate the significance p < 0.05.

Regression analyses stratified by gender yielded a different set of predictors of poor sleep quality in men and women with MS. In men, the final model consisted of pain, depression, and anxiety after adjustment for age, disease duration, and EDSS (Table 3). The only variable significantly associated with poor sleep quality in men was pain (OR = 12.7, 95% CI: 1.9; 29.6). The variables significantly associated with poor sleep quality in women included depression (OR = 4.1, 95% CI: 1.3; 13.2) and anxiety (OR = 6.8, 95% CI: 2.4; 19.1) (Table 3).

**Table 3:** Factors associated with poor sleep in men and women; odds ratios (OR), 95% confidence intervals (CI) for (OR) and *p*‐values from binary logistic regression

	PSQI			
	Men		Women	
	Odds ratio (95% CI)	*p*‐value	Odds ratio (95% CI)	*p*‐value
Predictors				
Pain (SF‐36)	**12.7 (1.9; 29.6)**	**0.01**	1.3 (0.4; 4.2)	.65
Depression (HADS‐D)	9.1 (0.9; 65.1)	0.07	**4.1 (1.3; 13.2)**	**.01**
Anxiety (HADS‐A)	4.0 (0.6; 25.3)	0.15	**6.8 (2.4; 19.1)**	**.001**
Covariates				
Age	1.0 (0.9; 1.1)	0.79	1.0 (0.9;1.0)	.69
Disease duration	1.0 (0.8; 1.2)	0.86	0.9 (0.8; 1.1)	.51
Disability (EDSS)	0.9 (0.5; 1.8)	0.87	1.1 (0.7; 1.8)	.54

PSQI, Pittsburgh sleep quality index; SF‐36, short‐form health survey; HADS‐D, hospital anxiety and depression scale – depression subscale; HADS‐A, hospital anxiety and depression scale – anxiety subscale; EDSS, expanded disability status scale. Bold values indicate the significance p < 0.05.

## 4 Discussion

The aim of this study was to identify possible gender differences in factors related to poor sleep quality in patients with MS. The results of our study suggest that different conditions may contribute to poor sleep quality in women and men with MS. We found that depression and anxiety were factors associated with poor sleep quality in women, whereas pain was associated with poor sleep quality in men.

Anxiety and depression are known to be common among individuals with MS (da Silva, Vilhena, & Lopes, 2011; Bianchi, De Giglio, & Prosperini, 2014). In earlier studies, both conditions were found to be important factors contributing to poor sleep quality in MS patients (Vitková et al., 2014; Boe Lunde et al., 2012), although we found no studies presenting data about their impact on sleep quality in women and men with MS. Observations from previous studies conducted among the general population and other chronic conditions showed depression and/or anxiety to be specific contributors of poor sleep in women (Mallampalli & Carter, 2002; Palagini, Tani, & Bruno, 2014; Castro‐Sanchez, Mataran‐Penarrocha, & Lara‐Palomo, 2012). A recently published study by Palagini et al., (2014) showed that the presence of depressive symptoms was the main determinant of poor sleep quality among women with systemic lupus erythematosus. A similar finding was shown among women with fibromyalgia (Castro‐Sanchez et al., 2012). The results of our study are in line with the findings among other types of diseases (Palagini et al., 2014; Castro‐Sanchez et al., 2012) and support our expectation that in the MS population both conditions could be the main contributors of poor sleep quality in women.

Concerning the factors associated with poor sleep quality in men, the strongest association was observed with pain. Pain is a common symptom in MS and is reported by more than half of patients with MS (Vitková et al., 2014; Foley, Vesterinen, & Laird, 2013; Fernández‐Muñoz, Morón‐Verdasco, & Cigarán‐Méndez, 2015). Our observation that pain may worsen sleep more in men than in women with MS seems to be inconsistent with previous research in the general population or with other chronic diseases (Silveyra et al., 2009; Van Onselen, Aouizerat, & Dunn, 2013). Considering that the prevalence of pain in our sample was almost identical across the gender groups, our findings cannot be explained by a different prevalence of pain in women and men, as was found in several studies in the general population or other chronic diseases (Hadjimichael et al., 2007; Svendsen et al., 2003). Thus, we can hypothesize that other aspects of pain may have played a role in the association with sleep quality. For example, gender differences in the peak time and characteristics of pain were found in a study by Morin, Lund, & Villarroel (2000) in a sample of patients with postsurgical pain. Women reported the highest pain intensity during the day, whereas men experienced higher intensities of pain in the evening. This observation could explain a more dominant role of pain in influencing sleep in men than in women. Furthermore, we used a self‐reported pain scale and the research in this area indicates that men are less likely to self‐report pain. When they do self‐report they often have difficulty in explaining the extent of their real pain experience (Brooks‐Brunn & Kelser, 2000). Thus, it can be deduced that women may have rated low‐intensity stimuli without the potential to influence the sleep as already painful, whereas men have rated as painful only those which interfered with their sleep. However, before any conclusion can be drawn in this regard more robust studies are required that employ a more comprehensive MS cohort with a broader range of ages and degrees of disability and that are accompanied by objective measures of sleep quality.

### 4.1 Strengths and limitations

In our study we used international, frequently used, and carefully validated questionnaires to obtain the data. Furthermore, all recorded answers were personally checked during the interview with the patient to avoid any confusion and increase the credibility of the answers.

Some limitations of this study should also be mentioned. The study has a cross‐sectional design, which does not allow us to explore the causal pathways between the studied variables. Most of the variables were measured by means of self‐report questionnaires. However, these have been used in different cultural settings and properly translated. Besides self‐reported sleep, it would be interesting to include both self‐reported and objective measures of sleep disturbances such as polysomnograms to obtain a more comprehensive assessment of sleep problems. We measured overall sleep quality without evaluation of specific sleep disorders such as obstructive sleep apnea or restless leg syndrome, which are common in MS patients and might contribute to poor sleep quality. Majority of our patients has mild or moderate disability, so our findings cannot be extended to the most disabled patients. Despite the relatively high response rate, 28% patients refused to participate. Those patients had significantly higher EDSS score in comparison with participants, so we may assume that it was the higher disability which might have prevented them from participating in the study. Finally, we found different models for men and women, but only a statistically significant interaction with gender for pain. This may imply chance findings of gender differences in relatively small number of patients in our study, showing a need for future research with a larger sample.

### 4.2 Implications

This study suggests that there might be an association between pain, depression, and anxiety on self‐reported sleep quality that differs between men and women. We found a stronger association among depression, anxiety, and sleep quality in women than in men, in whom the presence of pain was the main predictor of poor sleep quality. Therefore, the handling of symptoms associated with MS may require a somewhat gender‐specific approach.

This study should be replicated with a larger sample to confirm our findings in which objective measures of sleep quality should be used. Even though the total EDSS score did not play a significant role in explaining the poor sleep quality in our sample, it would be interesting to assess whether specific functional system scores could be predictors of poor sleep quality. As previous research hypothesized that the relationship among pain, depression, anxiety, and sleep disorders might be bidirectional (Caminero & Bartolomé, 2011), it would be interesting to explore the causal pathways between those variables to improve the understanding of poor sleep quality in MS patients. Moreover, the analysis of different aspects of sleep quality and their relationships with pain, depression, and anxiety could shed the light into those complex relationships.

### 4.3 Conclusion

The results of our study suggest that there might be an association among pain, depression, and anxiety on self‐reported sleep quality that differs between men and women. Understanding gender differences in sleep might allow for better diagnosis, treatment, and eventually prevention of poor sleep. If our findings are confirmed in further studies with more representative and broad‐based MS cohort it may highlight the need to apply gender‐specific approaches to the treatment of sleep disorders.

## Conflict of Interest

The authors report no conflict of interest.
